# Herd immunity, vaccination and moral obligation

**DOI:** 10.1136/jme-2022-108485

**Published:** 2023-06-05

**Authors:** Matthew Bullen, George S Heriot, Euzebiusz Jamrozik

**Affiliations:** 1 Box Hill Hospital, Eastern Health, Melbourne, Victoria, Australia; 2 Department of Infectious Diseases, Melbourne Medical School, University of Melbourne, Melbourne, Victoria, Australia; 3 Ethox Centre and Pandemic Sciences Institute, University of Oxford, Oxford, UK; 4 Royal Melbourne Hospital Department of Medicine, University of Melbourne, Melbourne, Victoria, Australia; 5 Monash Bioethics Centre, Monash University, Melbourne, Victoria, Australia

**Keywords:** Ethics, Policy, Public Policy, COVID-19, Epidemiology

## Abstract

The public health benefits of herd immunity are often used as the justification for coercive vaccine policies. Yet, ‘herd immunity’ as a term has multiple referents, which can result in ambiguity, including regarding its role in ethical arguments. The term ‘herd immunity’ can refer to (1) the herd immunity threshold, at which models predict the decline of an epidemic; (2) the percentage of a population with immunity, whether it exceeds a given threshold or not; and/or (3) the indirect benefit afforded by collective immunity to those who are less immune. Moreover, the accumulation of immune individuals in a population can lead to two different outcomes: elimination (for measles, smallpox, etc) or endemic equilibrium (for COVID-19, influenza, etc). We argue that the strength of a moral obligation for individuals to contribute to herd immunity through vaccination, and by extension the acceptability of coercion, will depend on how ‘herd immunity’ is interpreted as well as facts about a given disease or vaccine. Among other things, not all uses of ‘herd immunity’ are equally valid for all pathogens. The optimal conditions for herd immunity threshold effects, as illustrated by measles, notably do not apply to the many pathogens for which reinfections are ubiquitous (due to waning immunity and/or antigenic variation). For such pathogens, including SARS-CoV-2, mass vaccination can only be expected to delay rather than prevent new infections, in which case the obligation to contribute to herd immunity is much weaker, and coercive policies less justifiable.

## Introduction

Herd immunity is often invoked as an ethical justification for vaccination and/or coercive vaccine mandates.^1–3^ However, the term ‘herd immunity’ has multiple referents,^4^ which can result in ambiguity, including regarding the role of herd immunity in ethical arguments and policy debates. For example, ‘herd immunity’ can refer to (1) the herd immunity threshold (HIT) at which models predict the decline of an epidemic, sometimes eventually resulting in elimination; (2) the percentage of a population with immunity, whether or not it exceeds a given threshold; or (3) the indirect benefit afforded by (the sum of) individual immunity to non-immune people (also known as a ‘herd effect’).^5^

The accumulation of immune individuals in a population (herd immunity in the second sense above) can lead to two different outcomes. First, for pathogens such as measles or smallpox where immunity from infection or vaccination is highly effective at preventing (re)infection (sometimes referred to as ‘sterilising’ immunity), accumulation of immune individuals can result in elimination, that is, the sustained reduction of local transmission to zero. Maintaining R, the average number of secondary cases per infection, below 1 by keeping the immune fraction above a particular ‘herd immunity threshold’ (herd immunity in the first sense), is often understood to be a necessary condition for elimination in such circumstances.^6^

Second, for pathogens where immunity from infection or vaccination is relatively ineffective at preventing subsequent (re)infection, accumulation of immune individuals results in the development of an endemic equilibrium.^7^ Rather than being eliminated, such pathogens continue to circulate, often mutate, and (re)infect members of the population whose immunity wanes over time; examples include respiratory syncytial virus (RSV), influenza viruses, seasonal coronaviruses and SARS-CoV-2.^7–9^ The absence of elimination should not be confused with the absence of herd immunity, and indeed the term ‘herd immunity’ is often used to describe the accumulation of immunity in a population due to endemic viruses.^8^

In the endemic equilibrium case, herd immunity (in the second sense) nevertheless produces important benefits. For example, average severity of disease will be lower due to the sum benefits of individual immunity. This is what makes the difference between the high total mortality in the first wave of a pandemic (eg, COVID-19) or epidemic (eg, smallpox in the newly colonised Americas)^10^ and the lower mortality in subsequent waves, even in the absence of vaccination.^11^ There may also be some indirect protection of non-immune individuals (herd immunity in the third sense above) but this is often a relatively minor phenomenon for many pathogens because reinfections of immune individuals, although asymptomatic or minimally symptomatic, are often transmissible.^12 13^

Ambiguities about the referent(s) of the term ‘herd immunity’ in a particular context have the potential to undermine the validity of ethical arguments, including those which inform policy decisions. Specifically, where it is claimed that individuals have a moral obligation to contribute to herd immunity this may be used to justify coercive policies (ie, if one is obligated to do something one is less entitled to not do it, and so coercion is more acceptable from an ethical standpoint). Since the meaning of a term is arguably partly determined by its use, and since usage may vary, our intention here is not to argue that one particular meaning of the term is correct and the others mistaken.^4^ However, we will analyse two case studies to show that (1) the extent to which one has a moral obligation to contribute to herd immunity depends on the meaning of the term herd immunity, (2) the claim that the benefits of reaching an HIT entail special moral obligations is often misguided, and (3) that moral obligations to contribute to herd immunity in an endemic equilibrium are weak or non-existent.

### Case 1: diseases eliminable by herd immunity

The concept of an HIT is attractive from a public health policy perspective and is used to set vaccination targets for certain diseases.^4^ It is often linked with the goal of eliminating transmission of a pathogen in a particular population and thus is primarily relevant for vaccines or diseases where immunity generally prevents (re)infection (‘sterilising’ immunity) and transmission to others. An example is measles vaccines—although postvaccination measles infections do (rarely) occur, the vaccine is highly effective at preventing transmission to others (see table 1).^14^ The basic account is as follows:

**Table 1:** Comparison between features of vaccines for measles and COVID-19

	Measles	COVID-19
Vaccine prevents severe disease.	Yes	Yes*
Vaccine prevents transmission.	Yes	No†
Indirect protection of non-immune individuals (‘herd effect’).	Yes	No†
Mass vaccination results in elimination via herd immunity.	Yes	No
Immunity after infection prevents reinfection/transmission.	Yes	No
Vaccine available for higher risk groups.	No (eg, infants)	Yes (eg, older adults)

*Severe disease is nevertheless more common after COVID-19 vaccination than after measles vaccination.

†Current vaccines for COVID-19 provide partial and transient reduction in infection and onward transmission; however, this effect is not sustained for long periods of time.

*Predicted HIT*

- The fraction of immune individuals at which models predict that R (the average number of secondary infections per case) will fall below 1.
- Simple models calculate this threshold based on 1-1R0, where R0 (the basic reproduction number) is the average number of secondary cases expected to be cause by introduction of the pathogen into fully susceptible population.

Since predictions are not always accurate, there is also the:

*Observed HIT*:

- The fraction of immune individuals at which R<1 in a given population in a given state at a given time.

One of the key public health benefits of maintaining R<1 is that, if this is maintained, the disease will be eliminated (ie, local transmission of infection will fall to zero).

In order to characterise individual moral obligations to contribute to this phenomenon, a few clarifications are needed. First, since no vaccine is 100% effective, if all immunity is from vaccination the percentage of individuals who need to be vaccinated to reach the HIT is higher than the HIT. Second, for many relevant pathogens, population immunity is a combination of postvaccination and postinfection immunity. In most cases, an individual with postinfection immunity provides at least as much public health benefit as an individual with postvaccination immunity.^15^

Third, the percentage of immune individuals being above the HIT will suppress R below 1 *under certain conditions*. R0, from which the HIT is calculated, is a descriptive statistic derived from observations of the early phases of *specific real world* epidemics.^16^ Although it is often (arguably incorrectly) described as a property of the pathogen, it is impossible to isolate the transmissibility of a microbe from the context of its hosts and environment (ie, the contexts in which real world epidemics occur).^16^ The observed R0 for any given pathogen thus varies in different populations, different methods for deriving R0 produce widely divergent values, and transmission patterns may vary over time.^17^

Standard HITs are derived from simplistic infectious disease models that assume, for example, random mixing (each person in a population is equally likely to have contact with another person) and the absence of seasonality. However, the percentage of immune individuals required to suppress R below 1 will be inaccurately estimated by such models insofar as infections cluster together (mixing is non-random) or environmental and behavioural changes take place in winter (seasonal changes affect transmission); either of these factors, among others, can cause an unaccounted-for increase in transmission.

Fourth, it would be a mistake to conflate reaching the HIT with elimination. Even if we assume the basic account above holds, reaching the HIT is not the point at which elimination occurs and infections disappear. Just above the threshold, R is slightly below 1. This means that an infected person will, on average, transmit infection to slightly less than one other person. In most epidemics in sizeable populations a large number of cases (thus often considerable harm) will continue to occur after R reaches 0.99, even if it is maintained below 1, although fewer cases will occur over time. Moreover, the progression from reaching the HIT to elimination is not guaranteed and may in some cases be hindered by factors including waning immunity, altered patterns of contact in the population and (re)introduction of (new) strains of the pathogen.^18^

#### Moral obligations to contribute to elimination via herd immunity

We assume that the primary source of moral obligations in the context of vaccine-preventable diseases is the reduction of harm (or risk, ie, the probability of harm) to others. Although some authors claim that one should contribute to herd immunity whether or not one’s individual contribution reduces harm,^3^ the ultimate source of moral obligation entailed by such claims is presumably that the collective benefit of herd immunity consists in the reduction of (the probability of) harm. Moral reasons involving avoidance of harm provide a basis for coercive policies insofar as they align with Mill’s harm principle that restriction of people’s freedom of action may only be justified to prevent harm to others.^19^ ^i^

This leads to questions about the degree to which specific vaccines prevent harm to others (and thus the degree to which coercion might be justified on the harm principle) and about whether the collective benefit of elimination entails an additional (special) moral obligation to contribute to herd immunity *over and above* one’s obligation to become immune in order not to infect those with whom one comes into contact. The answer to such questions partly depends on the meaning of ‘herd immunity’.

Consider the moral obligations of a non-immune person P in three different populations:

- In population L, the percentage of immune individuals is below the HIT and there are cases of the disease in the community.
- In population M, the HIT has been exceeded and there are still cases of the disease in the community.
- In population N, the HIT has been exceeded and the disease has been eliminated.

On the *threshold view*, there are special moral obligations for person P to become immune in population L but not M because of the benefits of reaching an HIT. This is because, on this view, R falls below 1 when the HIT is reached. If this is maintained, the infection will be eliminated from the population. However, the difference between populations L and M can be that between R=0.99 and R=1.01. Even if the HIT is true, it is not clear that this is a difference of much ethical significance, since in either case an infected individual will infect on average approximately one other person in the population. Incidence may remain similar in both populations, and only after a protracted period will a noticeable difference in infections be observed, provided conditions are stable over time.

A deeper problem is that the idea of a threshold does not reflect many of the complex realities of disease transmission. No well-defined threshold exists at a particular point in time and space such that one person is the difference between population L and population M; as such, there is no clear distinction between these populations on which to base an obligation.

More plausible might be a *scalar view* of moral obligation, according to which the strength of one’s obligation to become immune tracks the degree to which this would reduce risk to others. While it might not be possible to have a high level of certainty regarding all factors relevant to risk, individuals can still develop reasonable heuristics, for example, high community transmission will be correlated with a higher probability of infection and frequent contact with high-risk people will be correlated with harmful outcomes. On this view, one has a (slightly) stronger obligation to become immune in population L than in population M because the risk to others of remaining non-immune is (slightly) higher in the former than in the latter. On this view, too, the threshold *per se* is irrelevant.

Suppose instead, on the *elimination view*, one has a moral obligation to contribute to the elimination of a disease and associated harms. This might seem to capture an important aspect of the HIT—that maintaining immunity above the observed threshold will result in elimination. This view suggests that person P in both populations L and M has an obligation to become immune because elimination has not been achieved. Again, this makes moral obligation contingent on local risk regardless of whether the HIT has been exceeded. On this view, however, a non-immune person would no longer have a moral obligation to become immune once the disease is eliminated from the local population (eg, in population N). Or, such obligations may cease when the disease is eradicated worldwide (and is thus unlikely to be reintroduced to populations where it is eliminated) as illustrated by smallpox.^ii^

On each of these views, there is no clear link between the goal of reaching an HIT and moral obligations to become immune. However, the scalar view can explain why moral obligations might vary instrength at different levels of herd immunity because, for example, risks to others fall as an increasing fraction of people become immune. Where herd immunity refers to the indirect protection of vulnerable individuals by people with immunity, this entails a moral obligation to contribute to the reduction of risk for this group, although on the scalar view one might think that this obligation becomes weaker (though not absent) at high levels of herd immunity where the risk is small - although this arguably ignores overdetermination.^20^ More broadly, this view will partly turn on empirical estimates of risk to others, which might not track herd immunity in a purely linear manner,^20–22^ and these empirical aspects of the problem arguably warrant further investigation. In any case, the scalar view significantly deflates any special moral claim attributed to the HIT or elimination, even for diseases that are, in principle, amenable to elimination via herd immunity.

### Case 2: endemic equilibrium

Apart from elimination, an alternative outcome of the accumulation of herd immunity is some degree of endemic equilibrium. This equilibrium reflects the balance that arises between pathogen and population when naturally acquired or vaccine-induced immunity does not prevent infection and/or transmission of infection to others (ie, immunity is non-sterilising), even if it reduces individual risk of disease. Reinfection, or postvaccination infection, occurs because (1) immunity wanes over time (even if it was sterilising initially this is no longer the case after, for example, several months), and/or (2) new variants of the pathogen evolve (ie, there is antigenic variation) and previously acquired immunity is less protective against infection with new variants (though it may still prevent severe disease).^iii^

Under such conditions, (re)infection of previously exposed or vaccinated individuals is common and the pathogen will continue to circulate even in the context of universal vaccination or previous infection, meaning elimination through exceeding a calculated HIT is an impossible goal. Many pathogens demonstrate this pattern, including influenza viruses, RSV, seasonal coronaviruses and SARS-CoV-2.^7–9 23^

Endemic equilibria are dynamic, that is, unstable, in part because they can be disrupted by changes in population immunity or the pathogen. For example, population immunity can be eroded by the interruption of transmission of endemic pathogens for extended periods of time. This erosion of herd immunity (or the accrual of ‘immunity debt’) occurred, for example, for RSV as a result of public health measures adopted during the COVID-19 pandemic. As a result, larger-than-usual ‘rebound’ epidemics occurred when RSV was reintroduced to populations where previous years’ usual winter epidemics (and the accumlated herd immunity response thereto) had been suppressed.^24^ Similarly, endemic equilibrium can be disrupted by the evolution of new variants of a pathogen that are more able to infect individuals with some degree of immunity to previous variants.^25^

Remarkably, considerable intellectual and political effort was wasted during the recent pandemic in speculation about whether supposed HITs to COVID-19 had been ‘reached’ (either via infection or vaccination) and whether this would result in elimination of the virus.^18^ It has been recognised for decades that reinfection is a common phenomenon with endemic coronaviruses in humans and that this is partly explained by partial immune cross-protection between different coronavirus variants.^26 27^ Moreover, many experts predicted that vaccine-derived immunity would follow a similar pattern to postinfection immunity, preventing severe disease on (re)infection but not preventing infection or transmission.^iv^ Under these conditions, elimination via herd immunity is impossible.^18^

It is now widely recognised that SARS-CoV-2 follows this pattern (as summarised in table 1). Reinfections and postvaccination infections are common, becoming more frequent over time since last exposure or vaccination (suggesting waning of immunity).^13 28^ Analyses of related coronaviruses suggest that once an endemic equilibrium is established, a median timeframe for reinfection may be around 1–2 years.^29^ Vaccination or immunity from past infection reduces severity but does not provide durable protection against infection or transmission to others.^30^ More specifically, current COVID-19 vaccines have partial effectiveness against infection lasting up to several months, after which there is no residual protection against infection^31^ and no difference in transmission risk.^32^ It is therefore expected that SARS-CoV-2 will follow a similar pattern to viruses like RSV which is known to cause repeated reinfections and settle into an endemic equilibrium punctuated by annual seasonal epidemics.^8^

#### Moral obligations in endemic equilibrium

How should we understand moral obligations in an endemic equilibrium state where elimination cannot be achieved by herd immunity?

Under conditions similar to those presented by SARS-CoV-2, all living people will eventually be (re)infected by current and/or subsequent variants of the virus in question. Vaccination can delay infection by perhaps a few weeks or months, but it cannot prevent infection.^33^ Nor does vaccination reduce the probability of transmission of (postvaccination) infections to a significant degree.^32^ Under such conditions, to what extent do people have an obligation to get vaccinated in order to protect others?

There are several reasons to think that there is no, or at most a very weak, moral obligation to be vaccinated for the sake of others in an endemic equilibrium. Individual vaccination (or receiving an additional ‘booster’ dose of vaccine) can at most delay one’s next infection. Thus, as opposed to vaccines that largely prevent a vaccinated person from becoming infected and infecting others, vaccines that delay infection provide only a temporary reduction in the risks imposed on others—and vaccination alone cannot prevent individuals from imposing risks of infection.

At the collective level, mass vaccination temporarily ‘tops up’ herd immunity but likewise can only delay a high cumulative incidence of infections. Herd immunity is constantly waning and being replenished by (re)infections. The primary goal of vaccination programmes under such conditions may therefore be to vaccinate those at highest risk of severe disease, including, for example, older adults whose immunity has waned.

Non-immune (or less immune) people arguably have at most a very weak moral obligation to be vaccinated to contribute to herd immunity under such conditions. First, because any protection of others is partial and short lived. As suggested, insofar as vaccines temporarily delay infection, low-risk individuals likely to transmit infection to those at higher risk in the near future may have a weak obligation to get vaccinated, but this may need to be balanced against any risks posed to these low-risk individuals by each act of vaccination.^34^ Second, all people will eventually become infected and thereby contribute to the replenishment of herd immunity regardless of whether or not they have previously been vaccinated.

Third, because of overdetermination of the risk of infection. People face risks of infection from vaccinated and unvaccinated individuals, from those with more immunity and those with less or no immunity. Whether a given individual I_1_ gets vaccinated makes very little difference to whether (or the time at which) person P gets infected, assuming that P has contact with many other potentially infectious individuals I_2_, I_3_, I_4_, etc. Under this assumption, person P’s probability of infection approaches 100% over time (eg, perhaps over a period of around 1–2 years for endemic coronaviruses). One way of thinking about this is that their risk of infection is overdetermined, since each of many contacts with others is potentially sufficient to cause P to become infected.^22^ Under these conditions, it is difficult to justify a moral requirement for I_1_ to get vaccinated.

The only situation in which individual vaccination might make a material difference is where person P has contact with one or very few people; under such conditions it might make sense for those people to take special precautions before visiting. But here vaccination might not be the most important precaution, in part because it only temporarily reduces transmission of the relevant pathogen to others. Moreover, a person who is vulnerable to one respiratory virus is likely vulnerable to many so it is arguably more important not to visit vulnerable individuals while one is symptomatic (or likely to be asymptomatically infected) with any virus than it is to be vaccinated against one particular virus.

The observation that one has no strong moral reason to be vaccinated for the sake of others in an endemic equilibrium should not give way to nihilism about herd immunity for such pathogens. Herd immunity (in the second sense) is a powerful force in transforming a devastating initial epidemic into milder seasonal epidemics. Vaccination can reduce the *individual* risk of severe disease during this transformation, but over and above this individual benefit it provides minimal protection to others.

#### Other possible reasons to contribute to herd immunity

Some might think that there are other potential justifications for coercive vaccine policies.^v^ Such justifications might rest on claims that individuals are morally obligated to get vaccinated (and contribute to herd immunity in the second sense) in order to improve public health by taking reasonable steps to reduce their individual contribution to the burden of the relevant disease (and thereby, for example, flatten the curve of a particular epidemic). Alternatively, the promotion of vaccination as a social norm might be considered to be sufficient to justify mandatory policies. We consider each of these potential justifications below.

##### Flattening the curve

The *flatten the curve argument* contends that individuals are morally obligated to minimise their personal risk of infection and serious illness in order to reduce strain on the health system. This is an extension of the harm principle in that one could conceivably harm others by consuming a finite health resource and thereby depriving someone else of it—a risk that might be salient in public health emergencies such as large epidemics. However, *flattening the curve* (of healthcare-requiring infections over time) is subject to the same implications as other harm prevention arguments discussed above: its strength depends on local quantitative assessments of risk, and it provides much weaker reasons for people at low baseline risk of healthcare use to get vaccinated. In addition, the argument is time sensitive in that it applies only during the period between epidemic recognition and one seroconversion interval (the time between vaccination and protection from dieasse) prior to the peak epidemic demand on healthcare resources.^35^

##### Improving population health

The *population health argument* broadens the case even further, claiming that individuals are morally obligated to get vaccinated as long as the collective outcome is a net health benefit in the population, irrespective of the extent to which individual contributions are likely to reduce harm to others (eg, via short term reduction in peak healthcare demand). On this view, the collective benefit (i.e., less cases of symptomatic illness as a result of herd immunity in the second sense) might be thought to justify requiring that every individual in the population comply with vaccination.

While many people working in public health might support this view as a justification for policy in general, using the improvement of public health to justify (highly) *coercive* policy may lead to more troubling implications. For example, since reducing the burden of sexually transmitted infections (STI) would go a long way to improving public health in many societies, this argument might imply that it would be ethically acceptable to *mandate* that all eligible citizens engage in yearly STI checks, with fines or other punishments for those who fail to comply. Assuming that such conclusions are unacceptable, the goal of improving public health might provide ethical reasons for policies that promote or subsidise vaccination, but it would not provide support for coercive policies such as vaccine mandates. Moreover, in the context of diseases like COVID-19, where individual risk factors for severe disease are well known, the population health argument might provide strong reasons for older adults or those with comorbidities including obesity to get vaccinated (since they account for the majority of health burden caused by the disease) but much weaker reasons for young healthy people to get vaccinated.

In general, the population health approach need not view populations as homogeneous. As more is learnt about risk factors for vaccine-preventable disease (or vaccine side effects), more precise policies should focus the use of vaccines where they can be expected to produce the most benefit (and no unacceptable risks). Examples of such approaches are policies recommending vaccines based on group characteristics such as age, pregnancy, ethnicity, or travel plans. Where risk factors are well described, such ‘precision public health’ approaches arguably strike a better balance between benefits and risks than universal policies.^vi^

##### Upholding social norms

Another possible source of obligation might be the importance of acting in accordance with social norms. One might argue that individuals ought to be vaccinated to support the practice of vaccination in general, regardless of their individual risk to self or to others (and regardless of their contribution to herd immunity in any sense). By getting vaccinated, individuals are publicly supporting the vaccine in question and perhaps reinforcing the notion of vaccination as a routine social duty. However, since social norms can produce a range of ethically good or bad outcomes, compliance with or promotion of norms *per se* cannot be sufficient source of moral obligation, nor can such compliance be the justification of coercive policies. To be a source of positive moral reasons, a social norm must produce net (public health) benefits—in the context of vaccination, this means that the norm (eg, universal vaccination with a given vaccine) must reduce harm.

While social norms making vaccination the default often do produce (net) benefits, they can also produce little or no benefit or even net public health harm. An example of no benefit is the norm of routinely revaccinating young adults with tetanus vaccines after potential re-exposure to the pathogen.^36^ An example of harm is undermining trust in vaccines; this occurred, for example, when uptake of dengue vaccines among children was followed by revelations that some children faced significant risks as a result of these vaccines, resulting in major declines in trust in vaccination in relevant communities.^37 38^

## Conclusions

We have argued that the strength of moral obligations to contribute to herd immunity through vaccination will partly depend on how the term is interpreted, and that not all interpretations are equally valid or useful in a given setting. For pathogens that are in principle eliminable via herd immunity, individuals arguably have obligations to contribute to this phenomenon, but the strength of obligations of non-immune individuals might be thought to decline as herd immunity increases. In the case of endemic diseases like COVID-19, herd immunity is best understood as a dynamic equilibrium between the pathogen and population where transmission may be slowed but the pathogen cannot be eliminated through vaccination and/or natural immunity. Under these conditions infections can only be delayed rather than prevented, and so the net benefit to others of vaccination is much less, and coercive vaccine policies less justifiable as a result.

## Data Availability

Data sharing not applicable as no data sets generated and/or analysed for this study.

## Footnotes

Some alternative justifications for coercion are either indirectly related to harm avoidance (eg, the goal of reducing strain on the health system) or invoke other potential sources of moral obligation (eg, support for public health norms). These will be returned to later in the discussion.

Note that since samples of smallpox virus are maintained in high biosafety-level laboratories, there remains a small risk of reintroduction of this virus into the human population.

For simplicity, we leave out other possibilities such as a dose–response effect where immunity protects against low-dose exposure but not higher dose exposures.

See, for example: https://www.statnews.com/2020/05/22/the-world-needs-covid-19-vaccines-it-may-also-be-overestimating-their-power/

We thank an anonymous reviewer for raising these potential justifications.

In settings where population differences are unclear there might be a stronger case for universal vaccination provided there is evidence for (likely) group benefit; however, as more data on intrapopulation differences become available vaccine policy should be adjusted accordingly.
